# Genotype–phenotype correlation in alpha-thalassemia: predicting genetic subgroups via laboratory parameters

**DOI:** 10.3389/fmed.2026.1825410

**Published:** 2026-05-19

**Authors:** Musa Fares Alzahrani, Saud Alrsheed, Homoud Al Gadheb, Saleh Albanyan, Badraa Muharib, Ghazi Alotaibi, Sarah Sewaralthahab, Fatima Alshalati, Ibrahim Alrumaih, Ahmed Jamal, Farjah Algahtani, Aamer Aleem

**Affiliations:** 1 Department of Medicine, College of Medicine, King Saud University, Riyadh, Saudi Arabia; 2 Oncology Center, King Saud University Medical City, Riyadh, Saudi Arabia; 3 Oncology Center, Chair of Epidemiology and Public Health Research, Faculty of Medicine, King Saud University, King Saud University Medical City, Riyadh, Saudi Arabia

**Keywords:** alpha, alpha-thalassemia, deletional, genetic, genotype, mutational, thalassemia

## Abstract

**Objectives:**

Alpha-thalassemia is caused by deletional and/or mutational genetic abnormalities. Limited information exists in Saudi Arabia about the genotype–phenotype correlation. We asked if certain changes in laboratory parameters can predict the underlying genetic subgroup.

**Methods:**

All patients diagnosed with genetically defined alpha-thalassemia at King Saud University Medical City between 2017 and 2024 were included. Data were collected retrospectively. We hypothesized that there is no difference between the mutation and deletion groups in terms of complete blood count, hemoglobin electrophoresis parameters, or ferritin. Mann–Whitney U and independent samples *t*-tests were used for between-group comparisons. Fisher’s exact or chi-square tests were used for categorical variables. Two-sided *p*-values of <0.05 were considered statistically significant. A descriptive summary was conducted for all the other variables.

**Results:**

A total of 378 patients were identified with alpha-thalassemia, of whom 276 had deletion, 64 had mutation, and 38 had both mutation and deletion. Of the 340 patients included in the final comparative analysis, the median age was 27 years, and 216 were female. Higher indirect bilirubin (12.3% versus 4%, *p*-value 0.013), mean red cell distribution width (RDW) (18.8 versus 17.1, *p*-value of 0.005), and heterozygous genotype status (71.9% versus 57.5%, *p*-value 0.036) were seen in the mutation group. Hemoglobin H (HbH) detectability was higher in the mutation group than in the deletion group (1.63 versus 0.02, *p*-value <0.001). These differences in RDW % and HbH detectability held true even after adjusting for sickle hemoglobin (HbS) level, with *p*-values of 0.006 and <0.001, respectively. No statistical difference was found between the two groups in ferritin level.

**Conclusion:**

Alpha-thalassemia mutations create a distinct hematologic phenotype characterized by increased red cell size variability, indirect bilirubin, and HbH compared to deletions, supporting different pathophysiological mechanisms. These could serve as simple laboratory markers to help distinguish between alpha-thalassemia subtypes, supporting the hypothesis that mutations create a more severe hemolytic phenotype than deletions.

## Introduction

Alpha-thalassemia is a group of inherited hemoglobinopathies that has a wide range of laboratory and clinical manifestations (1–3). The behavior of the disease and its clinical phenotype depend on different variables, including the nature of its genetic abnormality (4). There have been more than 100 genetic forms identified in alpha-thalassemia (1, 5–7). Generally speaking, these genetic abnormalities can be divided into deletional, mutational, and combined forms. Alpha-thalassemia arises from defective synthesis of the *α*-globin chain due to the deletion or mutation of one or more of the four alpha-globin genes. Complete deletion of the four genes results in the most severe clinical phenotype, which leads to intrauterine fetal death unless intrauterine transfusions are conducted (8). The presence of one functional gene can lead to the development of hemoglobin H disease (HbH), which is usually associated with significant anemia that may require blood transfusion (9). The presence of two or more functional genes is usually associated with silent carrier states. Non-deletional forms have been shown to be associated with a severe phenotype (3, 10, 11). The most common form of non-deletional (i.e., mutational) variant is Constant Spring, which is particularly common in many Asian countries (1). In Saudi Arabia and the Gulf regions such as Bahrain, however, the most severe presentation of HbH disease is attributed largely to the homozygous genotype of the T-Saudi polyA signal mutation, also known as the genotype of (α^TSaudi^α/α^TSaudi^α) (12).

Alpha-thalassemia is particularly prevalent in the Mediterranean, sub-Saharan Africa, and parts of Asia, although the disease is now prevalent in many regions of Europe and America due to recent migrations (13). Saudi Arabia has a high prevalence rate of alpha-thalassemia that varies according to the geographic location. Mecca and the eastern regions have a higher prevalence of 6% than other parts of the country, such as the northern regions, which have a prevalence of 0.4% (14–17).

Recent evidence has provided us a better understanding of the molecular nature of alpha-thalassemia in Saudi Arabia, with the most frequent abnormality being the deletional form −α^3.7^ (62.3%), followed by α2^IVS1(−5nt)^ (20.7%) and the mutational form α2 polyA-1 (α2^T. Saudi^) (14.1%) (18–21).

Deletional and mutational forms are not biologically or clinically equal. Despite this, the majority of studies have failed to account for how these two groups can differ phenotypically, which has led to an important gap in the literature. This genotype–phenotype correlation remains a critical research gap that persists despite the well-documented genetic differences. The majority of studies in the literature combined the two groups without stratified analysis. The importance of highlighting the difference is especially relevant in high-prevalence areas and/or areas with diverse migrant populations. Failure to distinguish the genetic subtypes may lead to misdiagnoses, misclassification, and potentially the undertreatment of severe non-deletional cases or unnecessary interventions in milder deletional forms (22). Furthermore, having some prediction of phenotype could aid the genetic counseling for patients wanting to have children and may help guide genetic testing.

Even with the recent literature that has elaborated on the genetic and geographic variations in our region, the laboratory and clinical phenotype differences between deletional and non-deletional forms of alpha-thalassemia remain unknown. We herein present our study aiming to answer this important question and try to close gaps in the literature.

## Materials and methods

This is a retrospective analysis that was conducted on patients with genetically confirmed alpha-thalassemia identified from the genetic database at King Saud University Medical City (KSUMC) from January 2017 until August 2024. The genetic abnormality was classified as either deletion, mutation, or both. We hypothesized that there is no difference between the mutation and deletion groups in terms of complete blood count (CBC) results, hemoglobin electrophoresis parameters, ferritin levels, or blood transfusion requirements. The alternative hypothesis was that there is a difference between these two groups.

Variables collected included CBC parameters such as hemoglobin (Hb), mean corpuscular volume (MCV), red cell distribution width (RDW), mean corpuscular hemoglobin (MCH), and mean corpuscular hemoglobin concentration (MCHC), and hemoglobin electrophoresis parameters such as the percentage of hemoglobin A (HbA), hemoglobin A2 (HbA2), fetal hemoglobin (HbF), sickle hemoglobin (HbS), and hemoglobin H (HbH). We also collected data on ferritin levels, the prevalence of transfusion, splenomegaly, and splenectomy. Hemoglobin electrophoresis was performed using Sebia capillary zone electrophoresis. The diagnosis of alpha-thalassemia was based on genetic confirmation. The diagnosis was achieved by performing the reverse dot blot/strip assay methodology using a reverse-hybridization principle, typically employed by commercial kits to detect common alpha-thalassemia deletions and point mutations. The test can detect 21 pathogenic mutations found in alpha-thalassemia patients. The genomic DNA was extracted from white blood cells and amplified by alpha-globin genes 1 and 2 (*HBA1 & HBA2*) specific biotin-labeled primers. The hybridization of amplification products onto a test strip containing allele-specific oligonucleotide probes immobilized as an array of parallel lines. Bound biotinylated sequences are detected using streptavidin–alkaline phosphatase and color substrates. The presence or absence of specific bands (lines) on the strip was interpreted according to the manufacturer’s instructions to determine the genotype (e.g., heterozygous or homozygous for a mutation).

A descriptive summary was conducted for all the other variables (proportions, median or mean, and range).

### Statistical analysis

Patients were classified into two primary genetic groups based on their alpha-thalassemia genotype: the deletion group, which included patients with deletion mutations only (≥1 deletion, 0 point mutations), and the mutation group, which included patients with point mutations only (0 deletions, ≥1 point mutation). Patients with both deletions and mutations (n = 76) were excluded from the primary analysis to ensure clear group comparisons.

#### Descriptive statistics

Continuous variables were assessed for normality using the Shapiro–Wilk test. Data are presented as mean ± standard deviation (SD) for all variables to facilitate comparison, despite some showing non-normal distributions. Categorical variables are presented as frequencies and percentages.

#### Comparative analyses

For univariate comparisons of continuous variables, the Mann–Whitney U-test was used for between-group comparisons (as the primary test due to non-normal distributions). Independent samples *t*-tests and Cohen’s d were used for effect size estimation.

For categorical variables, Fisher’s exact test was conducted for the primary analysis given small expected cell frequencies and the chi-square test.

### Multivariable analyses

Linear regression models were constructed for continuous outcomes. Hemoglobin was adjusted for age, sex, and the presence of HbS; MCV was adjusted for ferritin status (<50 vs. ≥ 50 μg/L) and the presence of HbS. Other CBC parameters (MCH, MCHC, RDW, and RBC count) and hemoglobin variants (HbA, HbA2, HbF, and HbH) were adjusted for the presence of HbS. Ferritin was also adjusted for age and sex.

Logistic regression models were used for binary outcomes as follows: transfusion history adjusted for age, sex, and HbS status. Low ferritin (<50 μg/L) adjusted for age and sex. Low MCV (<80 fL) adjusted for ferritin status and HbS presence.

Both univariate and multivariate odds ratios with 95% confidence intervals were reported. To test the hypothesis that low ferritin affects MCV differently in the deletion versus mutation groups, we conducted a linear regression analysis with an interaction term (genetic group × ferritin status) with pairwise comparisons using the Bonferroni-corrected pairwise *t*-tests.

All analyses were performed using Stata version 19 (StataCorp, College Station, TX). Two-sided *p*-values of <0.05 were considered statistically significant. No adjustment for multiple comparisons was applied to the primary outcomes, although a Bonferroni correction was used for post-hoc pairwise comparisons in the interaction analysis.

## Results

A total of 378 patients with genetically confirmed alpha-thalassemia were included in the study. Patients were categorized into three groups: the deletion group (n = 276) included patients with deletions only with no mutations, the mutation group (n = 64) included patients with mutations only with no deletions, and the mixed/other group (n = 38) included patients with both deletions and mutations. The primary analysis compared the 340 patients included in the deletion and mutation groups without patients with both abnormalities.

**Figure 1:**
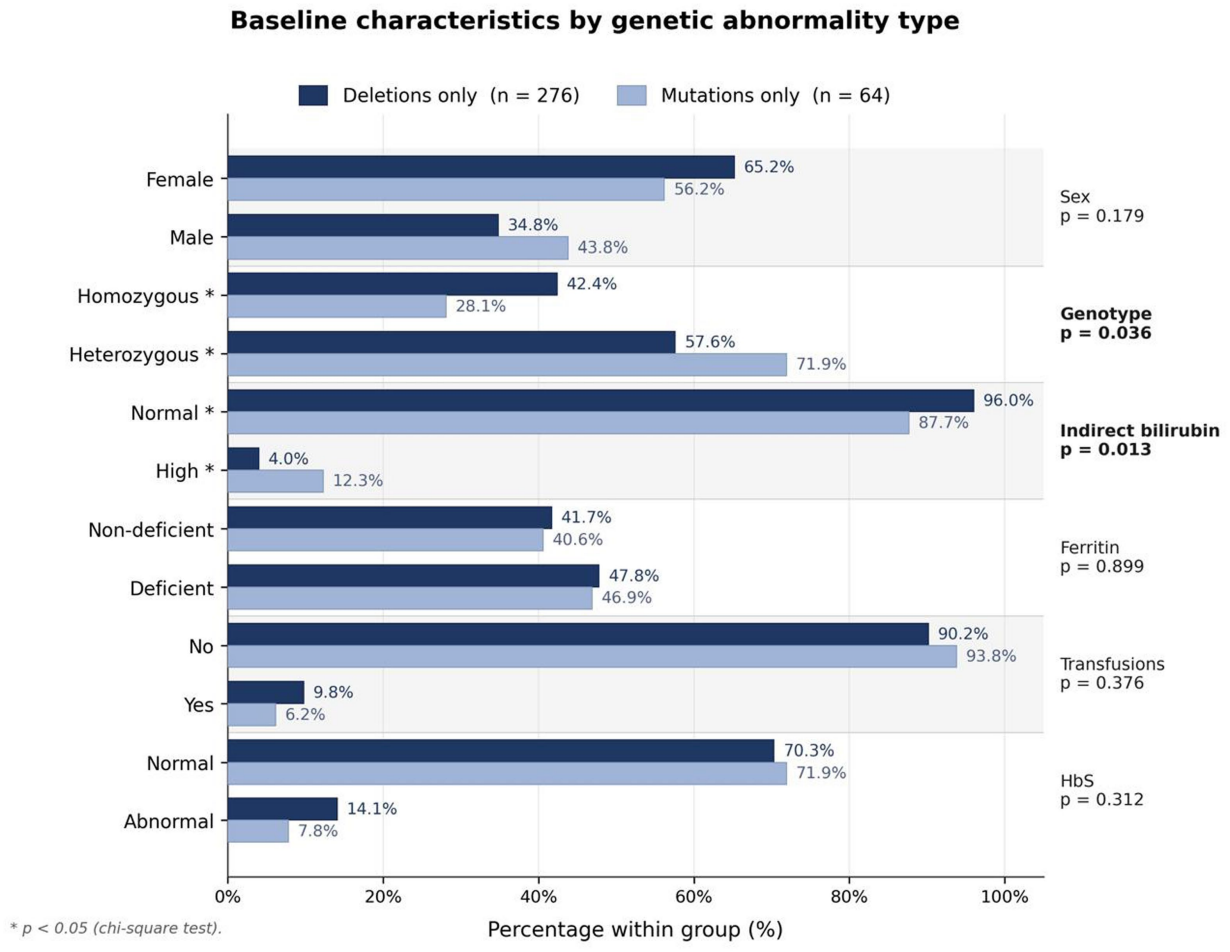
Baseline characteristics by genetic abnormality type. Comparison of deletions only (*n* = 276) versus mutations only (*n* = 64). *p*-values were calculated using the chi-square test, with asterisks (*) indicating statistical significance (*p* < 0.05).

Baseline demographics, including age, gender, and other clinical features, are shown in Figure 1 and are summarized in Tables 1–3. No difference was found in demographic data except for a higher heterozygous genotype status and higher indirect bilirubin in the mutation group. With regard to CBC parameters, a statistically significant difference was found in the median RDW %, which was significantly higher in the mutation group than in the deletion group (18.8 versus 17.1, *p*-value of 0.005). In the Hb electrophoresis parameters, the two groups differed in the median % of HbH, which was higher in the mutation group than in the deletion group (1.63 versus 0.02, *p*-value <0.001). HbH detectability was found in 10 of the 340 (2.9%) patients, of whom 2 of 276 (0.7%) were in the deletion group as compared to 8 of the 64 (12.5%) in the mutation group (*p*-value <0.001). These differences in RDW %, HbH %, and HbH detectability held true even after adjusting for HbS level (Table 4). No statistically significant difference was found between the two groups in transfusion history or ferritin parameters (Table 1). The most common genetic abnormality in our population was the –α^3.7^ single gene deletion, which was observed in 258 (68%) patients.

**Table 1:** Baseline characteristics by genetic abnormality type.

Variable	Genetic abnormality type			
	Deletions only	Mutations only	Total	Test
*N*	276 (81.2%)	64 (18.8%)	340 (100.0%)	
Age (years)	27.185 (19.86)	24.922 (17.79)	26.759 (19.48)	0.403
Sex				
Female	180 (65.2%)	36 (56.2%)	216 (63.5%)	0.179
Male	96 (34.8%)	28 (43.8%)	124 (36.5%)	
Genotype status				
Homozygous	117 (42.4%)	18 (28.1%)	135 (39.7%)	0.036
Heterozygous	159 (57.6%)	46 (71.9%)	205 (60.3%)	
Indirect bilirubin				
Normal	243 (96.0%)	50 (87.7%)	293 (94.5%)	0.013
High	10 (4.0%)	7 (12.3%)	17 (5.5%)	
Ferritin status				
Non-deficient (>50)	115 (41.7%)	26 (40.6%)	141 (41.5%)	0.899
Deficient (<50)	132 (47.8%)	30 (46.9%)	162 (47.6%)	
NA	29 (10.5%)	8 (12.5%)	37 (10.9%)	
History of blood transfusions				
No	249 (90.2%)	60 (93.8%)	309 (90.9%)	0.376
Yes	27 (9.8%)	4 (6.2%)	31 (9.1%)	
HbS status				
Normal	194 (70.3%)	46 (71.9%)	240 (70.6%)	0.312
Abnormal	39 (14.1%)	5 (7.8%)	44 (12.9%)	
NA	43 (15.6%)	13 (20.3%)	56 (16.5%)	

Data presented as mean ± SD for continuous variables and *n* (%) for categorical variables. Mann–Whitney U and independent samples *t*-tests were used for continuous while Fisher’s exact or Chi-square tests for categorical variables. N, number; NA, not available.

**Table 2:** Complete blood count parameters by genetic abnormality type.

Variable	Genetic abnormality type			
	Deletions only	Mutations only	Total	Test
*N* (%)	276 (81.2%)	64 (18.8%)	340 (100.0%)	
Hb: median (range)	11.30 (5.70–15.70)	11.25 (8.10–15.70)	11.30 (5.70–15.70)	0.568
RBC count	5.24 (0.92)	5.28 (0.79)	5.25 (0.90)	0.758
MCV	69.91 (7.68)	68.29 (8.45)	69.61 (7.84)	0.137
MCH	21.89 (3.09)	21.19 (3.41)	21.76 (3.16)	0.110
MCHC	314.14 (13.74)	312.38 (16.37)	313.81 (14.26)	0.374
RDW	17.09 (3.96)	18.77 (5.44)	17.40 (4.32)	0.005

Data presented as mean ± SD. *p*-values were calculated using the Mann–Whitney U test. *N*, number; Hb, hemoglobin; RBC, red blood cell count; MCV, mean corpuscular volume; MCH, mean corpuscular hemoglobin; MCHC, mean corpuscular hemoglobin concentration; RDW, red cell distribution width.

**Table 3:** Hemoglobin variants by genetic abnormality type.

Variable	Genetic abnormality type			
	Deletions only	Mutations only	Total	Test
*N* (%)	276 (81.2%)	64 (18.8%)	340 (100.0%)	
HbA	87.59 (23.55–97.50)	92.73 (11.02–97.30)	88.51 (21.92–97.50)	0.129
HbA2	2.65 (1.04–2.50)	2.38 (0.825–2.40)	2.60 (1.01–2.50)	0.084
HbF	1.40 (0.00–7.48)	0.57 (0.00–3.08)	1.25 (0.00–6.91)	0.437
HbH	0.02 (0.00–0.23)	1.63 (0.00–4.57)	0.31 (0.00–2.03)	<0.001

Data presented as mean ± SD and median. *p*-values were calculated using the Mann–Whitney U test. N, numbers; HbA, hemoglobin A; HbA2, Hemoglobin A2; HbF, fetal hemoglobin; HbH, hemoglobin H.

**Table 4:** Alpha-thalassemia adjusted analysis results.

Outcome	Adjusted for	Difference (95% CI)	*p*-value
Complete blood count parameters			
Hemoglobin (g/dL)	Age, sex, HbS	0.70 (−1.05 to 2.45)	0.433
MCV (fL)	Ferritin, HbS	−1.60 (−3.72 to 0.53)	0.140
MCH (pg)	HbS presence	−0.71 (−1.57 to 0.15)	0.103
MCHC (g/L)	HbS presence	−1.83 (−5.71 to 2.06)	0.355
**RDW (%)**	HbS presence	**1.67 (0.49 to 2.84)**	**0.006**
RBC count (×10^12^/L)	HbS presence	0.04 (−0.21 to 0.28)	0.768
Hemoglobin variants			
HbA (%)	HbS presence	1.95 (−2.73 to 6.64)	0.412
HbA2 (%)	HbS presence	−0.21 (−0.50 to 0.09)	0.166
HbF (%)	HbS presence	−0.66 (−2.76 to 1.43)	0.534
**HbH (%)**	HbS presence	**1.59 (1.00 to 2.18)**	**<0.001**

N, number; RBC, red blood cell count; MCV, mean corpuscular volume; MCH, mean corpuscular hemoglobin; MCHC, mean corpuscular hemoglobin concentration; RDW, red cell distribution width; HbS, sickle hemoglobin; HbA, hemoglobin A; HbA2, Hemoglobin A2; HbF, fetal hemoglobin; HbH, hemoglobin H. Bold values indicate statistically significant results.

A significant interaction was found between low ferritin and MCV, which indicates that the effect of low ferritin on MCV differs between genetic groups. Low ferritin substantially reduced MCV in the deletion group (*p* < 0.001) but had no significant effect in the mutation group (*p* = 0.707), supporting the hypothesis that mutations preserve MCV despite iron deficiency (Table 5).

**Table 5:** Ferritin-genetic subgroup interaction analysis.

Ferritin-genetic subgroup	*n*	Mean MCV ± SD (fL)	Low MCV <80 fL *n* (%)	*p*-value^1^
Deletion + Normal ferritin	115	72.2 ± 7.7	102/115 (88.7%)	Reference
**Deletion + Low ferritin**	132	**68.1 ± 7.4**	**128/132 (97.0%)**	**<0.001**
Mutation + Normal ferritin	26	66.2 ± 9.9	24/26 (92.3%)	0.018
**Mutation + Low ferritin**	30	68.7 ± 5.9	30/30 (100.0%)	0.707^2^

n, number; MCV, mean corpuscular volume; SD, standard deviation; fL, femtoliter. Bold values indicate statistically significant results.

**Figure 2:**
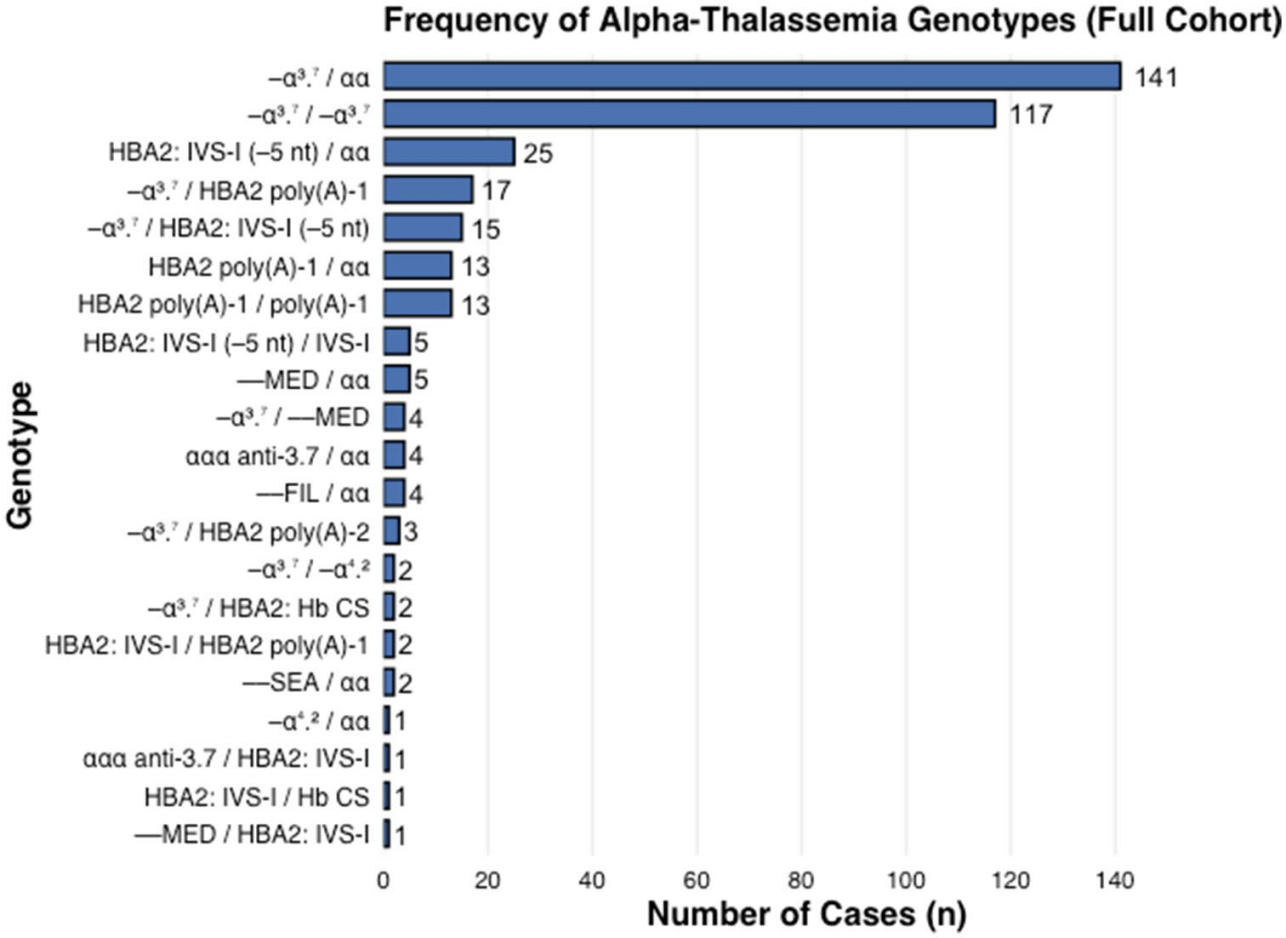
Frequency of alpha-thalassemia genotypes. This figure shows the number of cases (*n*) for each identified genotype within the full cohort. The single α-globin gene deletion (−α^3.7^ /αα) being the most frequent genotype (*n* = 141), followed by the homozygous deletion (−α^3.7^/−α^3.7^) (*n* = 117).

Figure 2 shows the frequency of different genotype subgroups identified in our study. More details about these genotypes are shown in Table 6.

**Table 6:** The different genotypes of alpha thalassemia in our study.

Genotype	Abnormality type	Description	N (%)
–α^3.7^/αα	Heterozygous	–α^3.7^ single gene deletion	141 (37%)
–α^3.7^/–α^3.7^	Homozygous	–α^3.7^ single gene deletion	117 (31%)
*HBA2*: IVS-I (−5 nt)/αα	Heterozygous	Single non-deletional point mutation in the first intervening sequence of *HBA2* gene	25 (7%)
–α^3.7^/*HBA2* poly(A)-1	Compound heterozygous	–α^3.7^ single gene deletion plus polyadenylation signal sequence Poly A1 (AATAAA > AATAAG); Saudi type	17 (4%)
–α^3.7^/*HBA2*: IVS-I (−5 nt)	Compound heterozygous	–α^3.7^ single gene deletion plus single non-deletional point mutation in the first intervening sequence of *HBA2* gene	15 (4%)
*HBA2* polyadenylation signal mutation – poly(A)-1/αα	Heterozygous	Polyadenylation signal sequence Poly A1 (AATAAA > AATAAG); Saudi type	13 (3%)
*HBA2* polyadenylation signal mutation – poly(A)-1/poly(A)-1	Homozygous	Polyadenylation signal sequence Poly A1 (AATAAA > AATAAG); Saudi type	13 (3%)
*HBA2*: IVS-I (−5 nt)/*HBA2*: IVS-I (−5 nt)	Homozygous	Single non-deletional point mutation in the first intervening sequence of *HBA2* gene	5 (1%)
−MED/αα	Heterozygous	Mediterranean double α-gene deletion	5 (1%)
–α^3.7^/––MED double gene deletion	Compound heterozygous	–α^3.7^ single gene deletion plus Mediterranean double α-gene deletion	4 (1%)
ααα anti-3.7/αα (anti-3.7 α-globin triplication)	Heterozygous	Alpha globin gene triplication	4 (1%)
––FIL/αα	Heterozygous	Filipino double α-gene deletion	4 (1%)
–α^3.7^/*HBA2* poly(A)-2	Compound heterozygous	–α^3.7^ single gene deletion plus poly A2 (AATAAA > AATGAA; Turkish type)	3 (1%)
–α^3.7^/–α^4.2^ (3.7/4.2 compound heterozygous single-gene deletions)	Compound heterozygous	–α^3.7^ single gene deletion plus –α^4.2^ single gene deletion	2 (0.5%)
–α^3.7^/*HBA2:* Hb Constant Spring	Compound heterozygous	–α^3.7^ single gene deletion plus hemoglobin Constant Spring mutation	2 (0.5%)
*HBA2:* IVS-I (−5 nt)/*HBA2* poly(A)-1	Compound heterozygous	Single non-deletional point mutation in the first intervening sequence of *HBA2* gene plus polyadenylation signal sequence Poly A1 (AATAAA > AATAAG); Saudi type	2 (0.5%)
−SEA/αα	Heterozygous	Southeast Asian double α-gene deletion	2 (0.5%)
–α^4.2^/αα	Heterozygous deletion	–α^4.2^ single gene deletion	1 (0.3%)
ααα anti-3.7/*HBA2:* IVS-I (−5 nt)	Compound heterozygous	Alpha globin gene triplication plus single non-deletional point mutation in the first intervening sequence of *HBA2* gene	1 (0.3%)
*HBA2:* IVS-I (−5 nt)/Hb Constant Spring	Compound heterozygous	Single non-deletional point mutation in the first intervening sequence of *HBA2* gene plus hemoglobin Constant Spring mutation	1 (0.3%)
−MED/*HBA2:* IVS-I (−5 nt)	Compound heterozygous	Mediterranean double α-gene deletion plus single non-deletional point mutation in the first intervening sequence of *HBA2* gene	1 (0.3%)

N, number; α, alpha; HBA2, Hemoglobin alpha 2 gene; IVS-I, first intervening sequence (intron 1); MED, Mediterranean double α-gene deletion.

## Discussion

To the best of our knowledge, our study is one of the largest, if not the largest, cohorts of genetically confirmed alpha-thalassemia reported from a single center in our region.

Our study has reported that RDW elevation in the mutational group was 1.67% higher than that seen in the deletional group (*p* = 0.006), indicating greater red cell size variability in the mutational subtype, which is independent of HbS status. We have also shown that HbH is the strongest discriminator between the genetic types, as it is dramatically elevated in the mutational group, in which it was 1.59% higher than the deletional group (*p* < 0.001) with a very high odds ratio (unadjusted = 21.49 and adjusted for the presence of HbS = 1.59). Furthermore, our analysis has shown a unique finding that iron deficiency affects the MCV in the two genetic types differently, suggesting distinct underlying physiologic pathways. Patients with low ferritin and the deletional type had a statistically significant difference in the mean MCV as compared to those with normal ferritin (68.1 versus 72.2, *p*-value = <0.001), but the same difference was not observed in the mutational group (68.7 versus 66.2, *p*-value = 0.707).

The most common genetic forms in our study were consistent with previous reports, with −*α*^3.7^ being the most common deletion and the IVS1 mutation being the most common mutation (18, 23). Alhuthali et al. reported that the most frequent abnormalities of alpha-globin genes were −α^3.7^ (62.3%), followed by α2^IVS1(−5nt)^ (20.7%) and α2 polyA-1 (α2^T. Saudi^) (14.1%). In the same study, it was interestingly shown that the Turkish mutation, also known as α2 polyA-2 (α2^T. Turkish^), can be identified in the Saudi population, and it can lead to HbH disease when it is present in a compound form with −MED (18). Our study included three patients with the Turkish mutation, but it was inherited in combination with the –α^3.7^ single gene deletion and did not result in hemoglobin H disease.

The genetic composition of alpha-thalassemia in the Saudi population is unique (19). For example, alpha-globin genes are commonly seen as one of two types (*HBA2* and *HBA1*), while in Saudis, there are three types, namely *HBA2*, *HBA1*, and *HBA12*. In addition, the poly A mutation [AATAAA>AATAAG] is very prevalent in Saudis, for which the name of Saudi poly A mutation was given. Moreover, co-inheritance with the sickle mutation or other beta thalassemia mutations is common in the Saudi population, which leads to variable genotype–phenotype presentations. Further studies are needed to help correlate the clinical and phenotypic manifestations of these genetic combinations.

Unlike our findings, one study from China concluded that patients with deletional genotypes of α-thalassemia were found to have higher RBC counts and lower Hb, MCV, MCH, and HbA2 than patients with non-deletional genotypes of α-thalassemia (*p* < 0.05) (24).

In our region, there is a paucity of data on the clinical and laboratory differences seen between mutational and deletional types of alpha-thalassemia. In one study from Bahrain, the most common form of HbH disease was related to mutational homozygous states rather than the prototypical deletional form of HbH disease, which was seen only in the minority of cases (12). Noteworthy, the genetic composition of HbH disease can be different from the composition of all alpha-thalassemia cases. For example, although -α^3.7^ is the most common genetic abnormality causing alpha-thalassemia in Iran, another study that included 120 cases with HbH disease showed that only 35 cases (29%) had the deletional form of HbH disease, while 71% had different forms of deletional/non-deletional and non-deletional forms. In this Iranian study, the non-deletional form was observed to be more severe than the deletional form, requiring more blood transfusions (25, 26). More studies from other countries in the Middle East, such as Saudi Arabia, are needed to highlight the difference in genetic composition between HbH disease and non-HbH disease alpha-thalassemia. Notably, the diagnosis of HbH disease is not always straightforward, and we did not specifically look at this particular subgroup in our limited study.

Our findings illustrate clear trends in genotype–phenotype correlation that could support future research and clinical practice. Many clinical scenarios could benefit from recognizing such correlations, such as dealing with a clinical phenotype that is more or less severe than expected or during antenatal genetic counseling.

The clinical relevance of our observations extends beyond research to routine laboratory diagnosis and patient management. In everyday hematology practice, CBC parameters combined with HbH detectability from hemoglobin electrophoresis can serve as practical “red flags” promoting expedited molecular genotyping to distinguish deletional forms from mutational forms in clinically relevant situations, especially in resource-limited settings where genetic testing is not universally available.

Our analysis has several limitations and could suffer from bias related to retrospective chart review studies. Given the multiple genetic subgroups that could have considerable phenotypic overlap, the results should be interpreted with caution. Thus, generalizing the study findings to the various genetic subtypes and their potential effects on laboratory parameters may need dedicated studies in the future. Moreover, the study hypothesis did not include a comprehensive list of other laboratory tests, such as hemolytic markers. However, we opted to limit the statistical analysis to a few variables to avoid the risk of multiplicity.

Despite these limitations, our study has many strengths. Our study has included a relatively large number of cases, all of whom were diagnosed genetically. In addition, our study has added information on the different types of genetic aberrations in our region, where limited information exists on the phenotype–genotype correlation. Finally, our study could help serve as a benchmark for future larger studies that can focus on building statistical prediction models to help predict the genetic subtype.

## Conclusion

Mutational alpha-thalassemia has a hematologic phenotype that is different from deletional types, which is characterized by increased red cell size variability, elevated indirect bilirubin, and higher HbH levels.

## Data Availability

The raw data supporting the conclusions of this article will be made available by the authors, without undue reservation, to any qualified researcher.
